# Influence of back support shape in wheelchairs offering pelvic support on asymmetrical sitting posture and pressure points during reaching tasks in stroke patients

**DOI:** 10.1371/journal.pone.0231860

**Published:** 2020-04-21

**Authors:** Atsuki Ukita, Masayuki Abe, Hirotoshi Kishigami, Tatsuo Hatta

**Affiliations:** 1 Social Medical Corporation Hokuto, Tokachi Rehabilitation Center, Obihiro, Japan; 2 Tohoku Fukushi University, Sendai, Japan; 3 Japan Health Care College, Eniwa, Japan; Northumbria University, UNITED KINGDOM

## Abstract

Many poststroke hemiplegic patients have an asymmetrical wheelchair-sitting posture. This study aimed to investigate the impact of different back support shapes on asymmetrical sitting posture and pressure points among poststroke hemiplegic patients during an activities of daily living–related reaching task. This study included 23 poststroke hemiplegic patients who performed tasks that involved the movement of objects using the unaffected upper limb to the affected side while sitting in a conventional wheelchair (C-WC) with a flat back support or a wheelchair providing pelvic and thoracic support (P-WC). Body alignment angles from video images and pressure distribution on supporting surfaces were measured using a two-dimensional motion analysis software (Dartfish) and a pressure mapping system (FSA). Regarding movement performance, although postural asymmetry increased in both wheelchair types, the degree of postural variation was smaller with P-WC use than C-WC use (p < 0.05), with partly reduced postural asymmetry. With P-WC, one-sided ischial asymmetrical pressure was significantly less after the movement (p < 0.05). In conclusion, P-WC’s back support shape contributed to a decrease in postural asymmetry for pelvic girdle support both at rest and during movement. This highlights the importance of a wheelchair back support shape and may help to increase the quality of activities of daily living movement in poststroke hemiplegic patients in wheelchairs.

## Introduction

Stroke is a major cause of death and disability in many countries. An estimated 7.0 million Americans experienced a stroke from 2013 to 2016, and 795,000 people experience a new or recurrent stroke each year [1]. Stroke is also a major health care problem in South, East, and Southeast Asia including in Japan [2]. According to a previous study in Japan, the incidence rate of overall stroke was 192.8 per 100,000 person-years and the estimated annual number of new strokes was 220,000 according to 2011 data [3], with about 1 in 5 middle-aged individuals showing a risk of experiencing stroke in their remaining lifetime [4].

Many stroke patients use wheelchairs during hospitalization while regaining the ability to walk; however, approximately 40% to 70% continue to use wheelchairs after they are discharged [5,6]. This indicates that stroke survivors use wheelchairs for a long time including during their hospitalization. In Japan, most wheelchairs in hospital or nursing facilities have a conventional back support (BS) shape (C-WC). The C-WC wheelchair type, involving a flat support surface without postural support and a straight BS post, is lightweight, inexpensive, and widely employed temporarily. However, although easy to prescribe, individual adjustments in C-WC are difficult and it is not suitable for use by patients with postural problems [7].

Stroke patients have complex imbalance characteristics [8–11] and asymmetrical postures are frequently observed among these individuals [12] during gait [13], standing [14], or when seated [15,16]. Asymmetric posture relates to an increase in spasticity [17] or weight unilaterally [11,18], so occurrence of this phenomenon in subacute stroke patients could hamper their progress toward recovery. Correcting the asymmetry is one of the aspects of rehabilitation [15,19,20] designed to improve patients’ activities of daily living (ADLs).

On the other hand, stroke patients’ less symmetrical posture during wheelchair-sitting may related not only to their imbalance characteristics but also the wheelchair’s shape itself. One study suggested that different positioning of the wheelchair seat and back affected the lumbar–pelvic angle and dynamic movement [21]. Additionally, variable thoracic or lumbar or pelvic (PL) support can generate different changes in muscle activation or pressure distribution or postural alignment, respectively [22–24]. This indicates that the shape or setting of wheelchairs also changes the posture or movement of users.

Focusing on the shape of C-WC, a previous study suggested that the contact area between the wheelchair and back (i.e., trunk including pelvis) was poor both in stroke patients and healthy adults [25,26]. The pelvis is the foundation of sitting posture and affects trunk positioning and lumbar and cervical spine alignment [27–30]. Additionally, it is indicated that proper trunk stabilization improves the ability to conduct upper-extremity functions [31,32]. In a wheelchair user, upper-limb movement is necessary to perform different ADLs [33,34].

Therefore, especially in patients with postural problems, a certain shape or set-up of the wheelchairs ensuring PL stability may be essential for the user to be able to maintain a seated posture and accomplish daily activities alongside promoting recovery from their complex imbalance characteristics. However, few studies have examined the effects of PL support when conducting dynamic tasks or during ADLs among patients in the hospital who are wheelchair users.

The aim of this study was to investigate the effects of a new BS design with PL support on asymmetrical sitting posture and pressure points during ADL-related tasks in poststroke hemiplegic patients. We hypothesized that a BS shape with PL support provides a midline sitting posture and distributes pressure evenly in both static and postactive postures, with the degree of PL support provided contingent on postural control.

## Materials and methods

### Participants

Twenty-three subacute poststroke hemiplegic patients (8 men and 15 women; 11 with left hemiplegia and 12 with right hemiplegia) were recruited. The inclusion criteria were a medically stable physical condition, ability to understand instructions, and ability to maintain a sitting posture for more than 30 minutes. All eligible patients had good control in the unaffected extremity but also have the risk of fall or have not regained the ability to walk without equipment: i.e., each locomotion functional independence measure (FIM) score was lower than 6 points. The exclusion criteria were dementia affecting the judgment to agree to participation in the study, difficulty in maintaining a static sitting posture or performing object movement tasks because of unilateral spatial neglect, attention impairment, visual field defects, and ataxia or apraxia.

Disease severity was assessed using Brunnstrom staging [35] and the Trunk Impairment Scale (TIS) [36]. TIS has the same reliability and validity as the Trunk Functional Evaluation of Stroke classification scheme [36]. Although there are few studies that have reported the reliability and validity of Brunnstrom staging directly, it generally incorporates the Fugl–Meyer assessment or Chedoke–McMaster stroke assessment, both of which are confirmed to have high degrees of reliability and validity [37,38].

This study was approved by the appropriate institutional review board of Hokkaido University Graduate School of Health Sciences (approval no. 13–44) and all subjects provided written informed consent for inclusion.

### Wheelchairs

**Fig 1:**
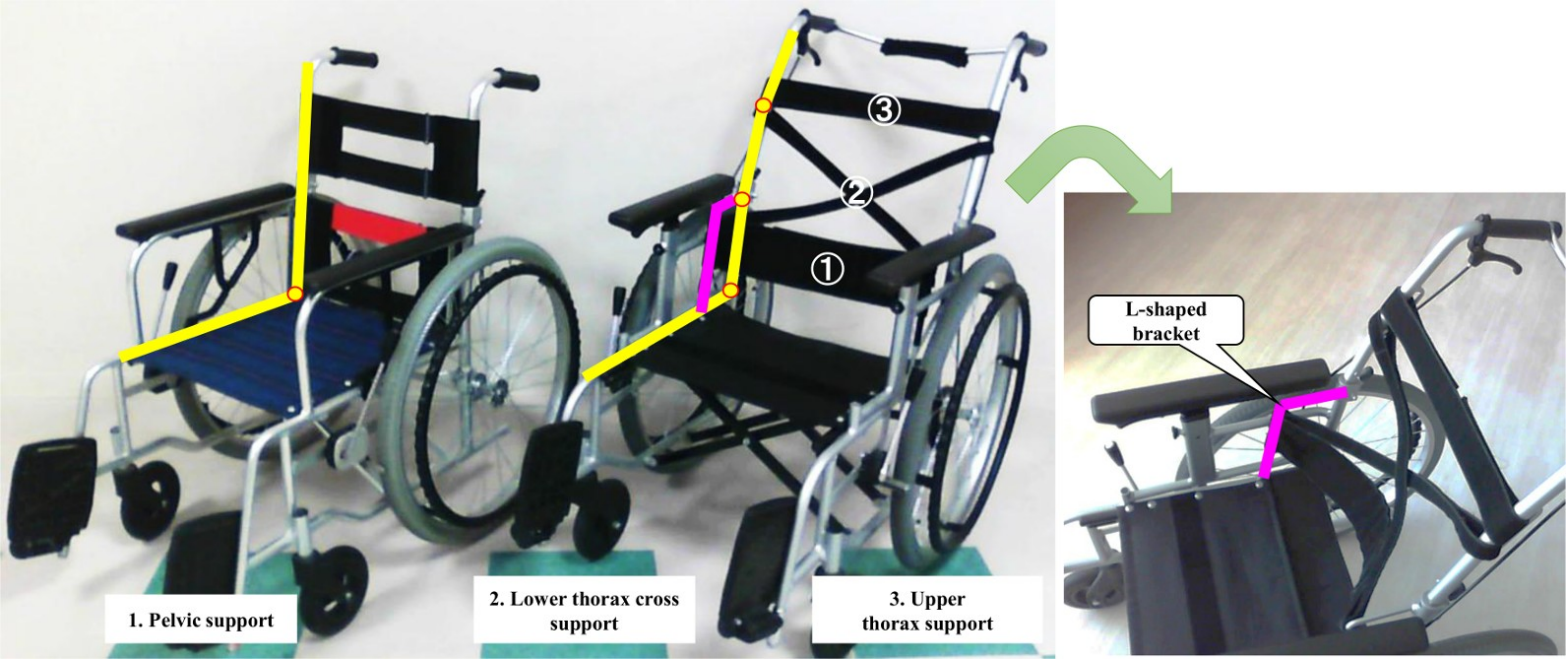
The difference in back support shapes. Left: C-WC. Right: P-WC. The sling seat of the back support was removed to unify the experimental conditions. The seat angles (i.e., the angle of orientation of the seat in the sagittal plane with respect to the horizontal plane as viewed from the side) were 2.9° for both wheelchairs. The post angle of the seat’s back support (i.e., the relative angle between the seat and the back support reference planes as viewed from the side) was 96° in C-WC, while the post angles of the seat’s back support in P-WC were 100° for PL, 110° for LT, and 121° for UT. The three belts can be set at several angles by changing the location of the fastener.

The present study used two wheelchair types, each with a 400-mm seat width. Both had a sling seat and a BS composed of flexible upholstery material(s) and tension adjustment for postural support. Fig 1 shows the different BS shapes of C-WC (NA406A; Nissine Medical Industries, Nagoya, Japan) and the wheelchair with PL and thoracic support (P-WC). P-WC (NA501A; Nissine Medical Industries, Nagoya, Japan) had three tension-adjustable support belts, and the BS posts were inclined backward in three stages. The three support belts in contact with the user’s back included the PL, lower thorax (LT), and upper thorax (UT).

The PL support belt involved two belts fixed at both ends of L-shaped brackets with hook-and-loop fasteners. The upper end of the PL belt was positioned at the iliac crest and the belt body was adjusted to support L4–L5; with this approach, pelvic support is realized from the side and back. The LT cross-belts run diagonally from the top of the PL belt to the lower end of the UT belt and support the thorax diagonally downward. The cross-belt intersection point is 1 inch (2.54cm) behind the PL belt. The UT belt was adjusted to avoid pressing into the scapula and thorax. On P-WC, the aforementioned tension adjustments were made before the experiment was conducted. The belts on C-WC were stretched tightly to reproduce a flat support surface.

### Experimental postural setting and procedure

Subjects were randomly seated in both wheelchair types. Physical and occupational therapists attached markers to each body segment after the subjects were seated. To minimize discomfort, a circular seal measuring 10 mm in diameter was used for facial attachment, and 12- to 20-φ retro-reflective markers (Nobby Tech. Ltd., Tokyo, Japan) were used at other locations. Markers were attached to the outer eye canthus, tragus of the ear, and C7 in the sagittal plane (only the nonparalyzed side) as well as the glabella, top of the chin, superior margin of the sternum, lateral tibial condyle, and both acromion processes in the frontal plane. After marker attachment, the subjects were instructed to sit normally and remain motionless with both hands placed on each thigh and both feet placed on the foot support [39,40]; this was defined as the preactive posture.

After assuming the preactive posture, a desk (720 mm in height) was placed in front of the subjects. A marking line to 60° from the midline (in front of the subject) was drawn on the desk. An object for movement (500-mL pet bottle) was placed on the desk on the unaffected side and the distance to the object was set within each subject’s upper-limb length.

**Fig 2:**
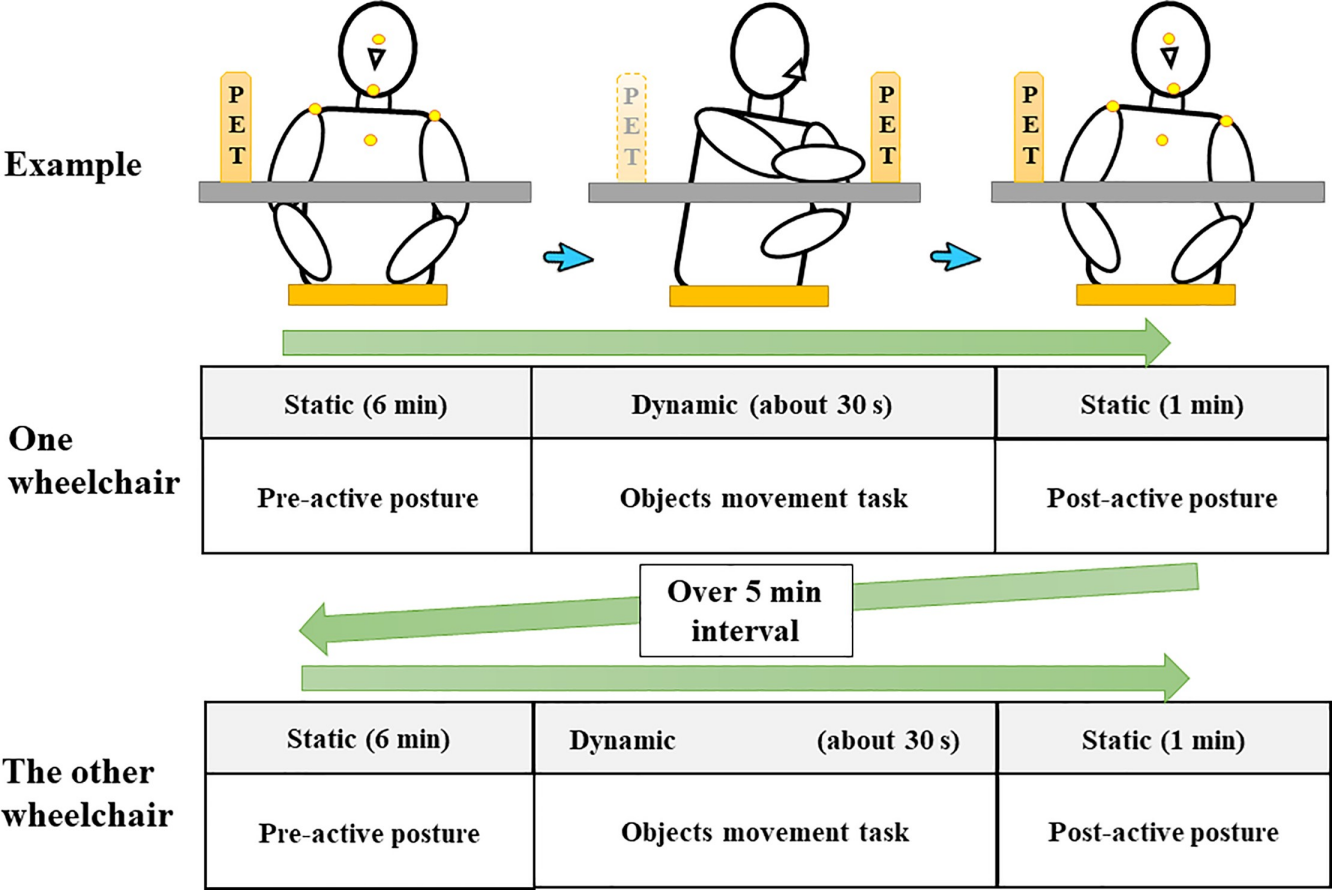
The overall procedure. A picture example of the task procedure (projected case #6; left hemiplegia) and highlighting of the marker placements. After finishing the task, both hands were replaced on the thighs and the subjects assumed the premovement posture again (postactive posture). Repositioning themselves during the experiment was prohibited.

The subjects were instructed to move the object as far as possible to the hemiplegic side on the marking line. They repeated this contralateral reaching task five times. In stroke patients, the trunk tilts to the hemiplegic side unconsciously [41] during object movement in the sitting position, and, therefore, this task is suitable for verifying real changes in posture. The overall procedure is shown in Fig 2.

### Postural alignment measurements

To measure postural alignment, the wheelchair-sitting posture was recorded in the sagittal (only the unaffected side) and frontal planes using a video camera. The height of the video camera was 720 mm and the distance from the camera to wheelchair was set at the minimum distance from the desk and wheelchair within the video frame. The video image was taken from 1 minute before to 1 minute after task initiation and completion.

Body alignment data from the markers on video were analyzed using a two-dimensional motion analysis software (Dartfish, Fribourg, Switzerland) previously used in postural assessment basic and clinical studies [42,43]. The analyzer system’s minimum angular resolution is 0.1° and the minimum distance resolution is 1 cm.

**Fig 3:**
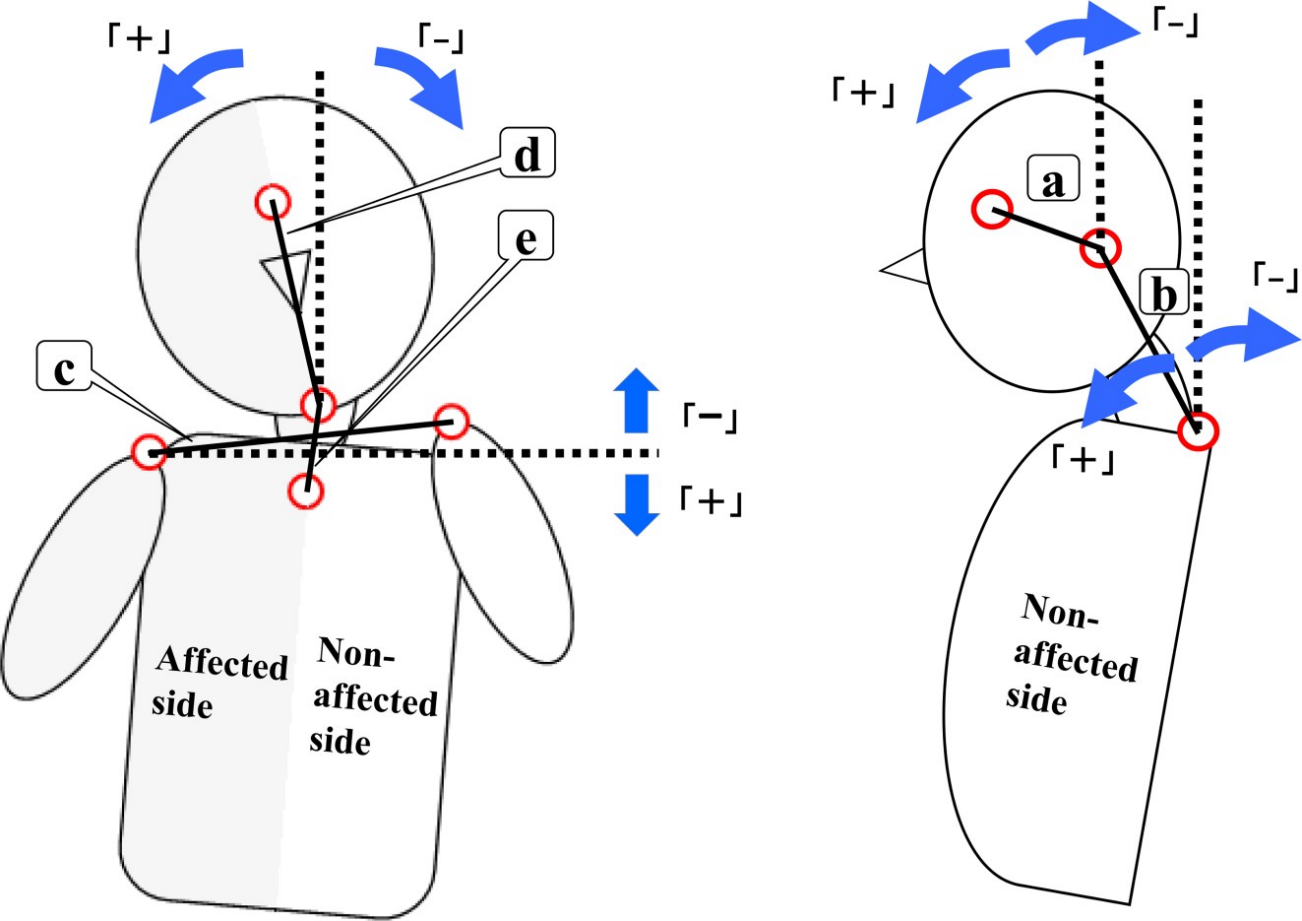
The criteria for angles and the direction of angular variation. a: Head inclination angle, between the vertical line and the line through the tragus of the ear and outer eye canthus. b: Neck inclination angle formed between the vertical line and the line through C7 and the tragus. c: Acromial tilt angle formed between the horizontal line and the line through both acromion processes. d: Head lateral inclination angle formed between the vertical line and the line through the glabella and the top of the chin. e: Neck lateral inclination angle formed between the vertical line and the line through the top of the chin and the superior margin of the sternum. The variation through the tasks was represented as postactive posture angle–preactive posture angle. This figure is an example of right hemiplegia.

Head inclination, neck inclination, acromion tilt, head lateral inclination, and neck lateral inclination angles were measured [40,44,45]. Each measured angle in the frontal plane was an absolute value representing postural asymmetry against gravity or horizontal lines. The body segment midline on the gravity line was represented as 0° for each angle. Fig 3 shows the angular definition and angular variation direction.

In the frontal plane, postangles smaller than preangles (i.e., correction of posture through movement) were represented as “minus” changes, while postangles larger than preangles (i.e., loss of posture through movement) were represented as “plus” changes.

In the sagittal plane, postangles smaller than preangles (i.e., more upright head or neck position through movement) were represented as “minus” changes, while postangles larger than preangles (i.e., more frontward head or neck position through movement) were represented as “plus” changes.

Changes in tragus and external condyle positioning from the markers were calculated. Anterior movements of the tragus and external condyle against the preactive posture were represented as “plus” changes, while posterior movements of the tragus and external condyle against the preactive posture were represented as “minus” changes.

Variations were extracted on one 10-second frame prior to and after the object movement task.

### Pressure measurements

A force sensing array (FSA, Vista Medical Ltd., Winnipeg, Canada) system was used to collect pressure data from the seat and BS interfaces. The FSA mat in the BS was also used as a BS cover. A web camera was synchronized to the FSA to identify movement start- and endpoints. Pressure distribution was taken from 30 seconds before to 30 seconds after movement initiation and completion.

FSA is commonly used for clinical pressure measurements [18,46]. The FSA mat has 256 sensors with a measured pressure range of 0–200 mmHg. FSA sampling rates were set at 5 Hz and calibrated before the experiment [47].

The maximum pressure, mean pressure, center of pressure (COP), and sensing area were automatically calculated using the FSA software version 4.0. If the COP point was placed on the center of the supporting surface, the FSA system represented a COP of 21.6 cm on both horizontal and vertical lines. From these values, total pressure (average pressure × sensing area/weight; mmHg*cm^2^/kg) at the back and seat and asymmetrical pressure were calculated. The asymmetrical pressure assessed differences in pressure distribution during sitting and the bilateral 3 × 3 pressure transducer data of the ischial tuberosity areas were processed. The asymmetrical pressure formula is shown below:

$$\begin{array}{l} asymmetrical\;pressure\;(mmHg)\\ \;=|(average\;pressure\;of\;non-affected\;ischial\;area)\\ \;-(average\;pressure\;of\;affected\;ischial\;area)| \end{array}$$

For asymmetrical pressure, postpressures smaller than prepressures (i.e., asymmetrical ischial pressure decreased) were represented as “minus” changes, while postpressures greater than prepressures (i.e., asymmetrical ischial pressure increased) were represented as “plus” changes.

To represent pelvic position displacement because of movement, the pelvic rotation distance was calculated from all movements of the bilateral 3 × 3 pressure transducer ischial tuberosity data. To represent pelvic rotation in the sagittal plane, the vertical COP change of both ischial tuberosities was used.

All pressure variations, except pelvic displacement, were extracted using the same angle measurement process.

### Statistical analysis

The Statistical Package for the Social Sciences version 22.0 for Windows (IBM Corp., Armonk, NY, USA), was used for data analysis. Static preactive postures between wheelchairs and variations of such throughout the task were compared. Because of the small study sample size, the Wilcoxon signed-rank test was used to compare data collected from the two wheelchairs, and p-values of less than 0.05 were considered to be statistically significant.

## Results

### Demographic data

Twenty-two patients (age: 71.5 ± 11.02 years; weight: 53.9 ± 11.35 kg; height: 155.3 ± 10.65 cm) were analyzed. One patient (female, with right hemiplegia) was excluded because she was seated in a C-WC without using the BS, defined as a relaxed posture.

The mean number of days from stroke onset was 96.4 ± 41.13 days. Brunnstrom’s staging results were averaged for the upper limbs (3.3 ± 1.29) and lower limbs (3.8 ± 1.15). The average total TIS score was 12.8 ± 4.09. Subjects’ individual profiles are shown in Table 1.

**Table 1:** Demographic and clinical data.

No.	Disease	Gender	Paretic side	Days since stroke onset	Brs (UE)	Brs (LE)	TIS
1	CI	F	L	71	4	4	15
2	CI	F	L	81	5	5	12
3	CI	F	L	126	1	2	15
4	ICH	F	L	103	2	2	6
5	CI	F	L	129	2	3	12
6	CI	F	L	178	4	4	12
7	ICH	M	L	97	2	2	10
8	CI	F	L	122	3	4	11
9	ICH	M	L	141	4	5	16
10	CI	F	L	41	4	4	18
11	CI	F	L	127	5	5	15
12	CI	F	R	83	2	4	9
13	CI	M	R	139	3	4	12
14	ICH	F	R	30	5	5	14
15	CI	M	R	151	2	2	16
16	ICH	M	R	59	5	5	16
17	CI	M	R	76	2	3	10
18	CI	M	R	43	4	4	16
19	ICH	F	R	32	5	5	19
20	CI	F	R	74	4	4	3
21	CI	M	R	95	3	5	17
22	CI	F	R	122	2	2	7

CI: cerebral infarction; ICH: intracerebral hemorrhage; L: left; R: right; Brs: Brunnstrom stage UE: upper extremity LE: lower extremity

### Postural alignment

Postural measurements are summarized in Table 2. For frontal-plane preactive postural alignment, there was a significant difference noted in the acromion tilt angle and it was smaller in P-WC than in C-WC (p < 0.05). For angle variation through the tasks, there were significant differences in acromion tilt and head lateral inclination angles. Although the head and shoulder alignment moved gradually away from neutral, the postural change for each was smaller in P-WC (p < 0.05). There were no angle differences in neck lateral inclination angle variation; however, the neck moved further from the midline in C-WC and was closer to neutral in P-WC.

**Table 2:** Measurements of postural alignments.

			Preactive alignment			Variation		
			C-WC	P-WC	p-value	C-WC	P-WC	p-value
			median (quartile)	median (quartile)		median (quartile)	median (quartile)	
**Angle (degrees)**	Frontal plane	Acromial tilt	2.2 (2.03)	1.1 (1.63)	< 0.05	+3.6 (3.53)	+1.3 (3.80)	< 0.05
		Head lateral inclination	3.1 (3.80)	3.2 (3.73)	ns	+1.7 (2.35)	+0.6 (2.60)	< 0.05
		Neck lateral inclination	4.2 (4.65)	4.5 (4.90)	ns	+1.8 (4.48)	−0.2 (4.20)	ns
	Sagittal plane	Head inclination	64.4 (6.35)	67.8 (7.95)	< 0.01	−1.4 (3.58)	±0.0 (3.50)	ns
		Neck inclination	44.9 (7.35)	37.6 (9.30)	< 0.01	+1.0 (3.78)	+0.7 (2.75)	ns

Variation (frontal plane): the value of how each alignment angle moves from the preactive posture against the midline

Variation (sagittal plane): the value of how each alignment angle moves back and forth against the preactive posture

Preactive postural alignment in the sagittal plane was significantly different for head and neck inclination angles and was smaller in P-WC than in C-WC (p < 0.01). In terms of angle variation throughout the tasks, there were no differences in either head and neck inclination angle variation; however, the final head and neck alignments were changed to a chin-up posture in C-WC.

Table 3 presents data on tragus and external condyle position displacement in the sagittal plane. The tragus final position moved backward in C-WC and forward in P-WC, respectively (p < 0.05), while the external condyle position moved forward in C-WC and remained the same in P-WC (p < 0.01).

**Table 3:** Displacement of marker.

		Variation		
		C-WC	P-WC	p-value
		median (quartile)	median (quartile)	
**Displacement (cm)**	Tragus	−1 (3.50)	+0.5 (1.00)	< 0.05
	External condyle	+0.5 (1.75)	±0.0 (1.00)	< 0.01

Variation: the value of how each position moves back and forth against the preactive posture

### Pressure

Table 4 summarizes basic pressure data. In the preactive posture, the BS demonstrated a large contact area and received more pressure (p < 0.01), with a lower seat pressure (p < 0.01) present in P-WC relative to C-WC. This trend did not change through the task.

**Table 4:** Basic pressure data.

		Preactive posture			Variation		
		C-WC median (quartile)	P-WC median (quartile)	p-value	C-WC median (quartile)	P-WC median (quartile)	p-value
**Maximum pressure (mmHg)**	Seat plane	200.0 (14.33)	200.0 (44.61)	ns	±0.0 (3.39)	±0.0 (13.01)	ns
	BS plane	78.3 (86.12)	127.8 (49.76)	< 0.01	+4.0 (33.58)	−13.9 (38.36)	ns
**Average pressure (mmHg)**	Seat plane	37.9 (14.62)	33.8 (7.81)	< 0.01	−0.3 (4.98)	−1.4 (5.82)	ns
	BS plane	19.3 (12.58)	20.9 (6.25)		+0.6 (9.72)	−1.0 (4.43)	ns
**Sensing area (cm^2^)**	Seat plane	1064.3 (295.25)	951.3 (233.28)		+10.9 (34.63)	+51.0 (58.32)	< 0.01
	BS plane	277.0 (147.62)	586.8 (174.94)	< 0.01	−32.8 (45.56)	−21.9 (107.53)	ns
Total pressure (mmHg*cm^2^/kg)	Seat plane	715.1 (150.10)	597.3 (16.81)	< 0.01	−23.8 (64.47)	+12.6 (109.89)	ns
	BS plane	93.3 (73.33)	249.7 (70.30)	< 0.01	−5.5 (60.06)	−25.4 (71.51)	ns

BS: Back support

Variation: the value of how each pressure moves against the preactive posture

Table 5 shows asymmetry pressure data. Each COP represented the distance from the mat’s center. In the preactive posture, all COPs were closer to the center of each support surface (p < 0.05), and the pressure bias around one ischial tuberosity was lower in P-WC relative to C-WC. Asymmetrical pressure variation through the task was increased in C-WC and reduced in P-WC (p < 0.05).

**Table 5:** Pressure data regarding asymmetry.

			Preactive posture			Variation		
			C-WC	P-WC	p-value	C-WC	P-WC	p-value
median (quartile)	median (quartile)	median (quartile)	median (quartile)					
**Center of pressure (cm)**	**Horizontal center**	Seat plane	1.1 (0.95)	0.6 (0.82)	< 0.05	+0.5 (1.56)	+0.7 (1.06)	ns
		BS plane	2.3 (1.77)	1.5 (2.31)	< 0.05	+1.4 (3.51)	+0.3 (1.85)	ns
	**Vertical center**	Seat plane	3.0 (2.31)	1.3 (1.99)	< 0.01	−0.1 (1.20)	+0.1 (0.94)	ns
		BS plane	6.3 (4.51)	2.8 (1.94)	< 0.01	+0.8 (3.15)	−0.3 (2.2 0)	ns
**Asymmetrical pressure (mmHg)**			31.2 (24.38)	24.3 (27.18)	ns	+11.2 (43.90)	−1.5 (28.04)	< 0.05

BS: Back support

Variation (center of pressure): the value of how COPs move from the preactive posture against the center of surfaces

Variation (asymmetrical pressure): the value of how one-sided pressure is increased from the preactive posture

Regarding the pelvic position, displacement through the task increased in both wheelchairs and the pelvis is rotated to the affected side. Displacement was 3.0 ± 4.38 cm in C-WC and 0.9 ± 1.96 cm in P-WC (p < 0.05).

## Discussion

We compared the wheelchair-sitting posture and pressure distribution of hemiplegic patients in C-WC and the novel P-WC with a unique BS shape. Patients performed the same task but showed different reactions between the two wheelchairs. The average TIS score of patients was 12.8 points, indicating lower trunk function than an ambulatory group (TIS: 14 points) or lower independence than an ADL group (TIS: > 20 points) [48,49]. This score indicated that these individuals now need to depend upon a wheelchair for their activities and that different results were produced during the same task regardless of the Brunnstrom’s stage on the paralyzed side.

Regarding preactive sitting posture, on the BS plane, the contact area between the wheelchair and patient, including the PL part, was increased and the COP was located nearer to the center of the supporting surface in P-WC than in C-WC. These results support the outcomes of our previous studies involving both stroke patients and healthy adults in static experiments [25,26]. The P-WC BS post is inclined backward in three stages, with a 100°-wide angle design in the PL region as compared with a 96°-wide angle design in C-WC. The post angle and each P-WC support belt provide support diagonally downward to receive the weight of the trunk, acting unlike a simply flat support surface seen in a typical reclining wheelchair BS, and the contact area expansion leads to an increase in the tactile sensation on each segment of the back. After stroke, reduced stability and symmetry are observed in the thorax and pelvis [50]. Further, postural perception is derived from graviceptive–somaesthetic information, given that head orientation is mainly supported by the vestibular information, whereas trunk orientation is supported by the somatosensory information [51,52]. As such, the supportive wide contact area of the BS shape facilitates lateral stability and increases the tactile sensation, contributing to COP movement to the center of the supporting surface. In the P-WC seat plane, average and total pressures were decreased. Moreover, pressure was more central and asymmetrical pressure was smaller in P-WC than in C-WC. The lateral support by the P-WC BS shape also affects the seat plane with a reduction of intensive pressure to one side provoked by correcting the positioning of the trunk.

Through the dynamic task, the stroke patients could not maintain a midline sitting posture in both wheelchairs; however, alignment variation was smaller in P-WC than in C-WC.

In C-WC, postural alignment changed to almost a sliding posture with PL rotation toward the paralyzed side with neck flexion. The straight BS shape further worsens the condition of stroke patients during the task; this sliding posture is disadvantageous for natural upper-limb operations with trunk forward movement with respect to the pelvis [53,54]. Moreover, this forward head posture is considered to hamper activities, e.g., eating or swallowing [55]. In the seat plane, asymmetrical pressure in C-WC increased through the task. In the study of pressure mapping of the seat surface, lower pressure was observed when using a custom-wrought cushion than the planer surface [56,57]. The seat shape affects pressure mapping, but the seated pressure also changes depending on the user’s posture [58] and, either way, a high-pressure distribution of ischium promotes a risk for the onset of a pressure ulcer [59]. A cross-sectional study involving 571 patients in long-term units suggested a high-risk relationship between pressure ulcer and poststroke status existed more strongly than other positive association risk factors (e.g., trauma or cognitive decline) [60]. Additionally, stroke patients are not good at repositioning themselves and it is preferable to avoid increasing asymmetrical pressure on the buttocks through movement.

In contrast, P-WC users experienced an asymmetrical reduction in the neck lateral inclination angle and asymmetrical ischial pressure. The P-WC PL support belt is flexible and follows the PL movement in maintaining supportive function. Therefore, P-WC contributes to continuous pelvic support during the task in accordance with PL position and shape changes. These PL support characteristics suppress buttock displacement during the task. Continuous pelvic support provides a stable uninterrupted postural foundation and has the advantage of providing uninterrupted tactile information from the PL region during movement. Somatosensory system plasticity is utilized in stroke rehabilitation to treat asymmetry [61,62]. Unlike the poor PL support seen with C-WC, the P-WC shape contributes to an asymmetry reduction by maintaining PL stability throughout the task.

### Limitations

The generalizability of these results may be limited by the small study sample size and the present results only verify the immediate effects of one simple ADL task, i.e., lateral movement of an object. In addition, all subjects were of standard body proportions; therefore, these findings may not be generalizable to populations with different anthropomorphic parameters. Also, only a two-dimensional analysis was performed; therefore, there were no rotation (convolution) angle data collected. Moreover, regarding asymmetry, the measurement results were absolute values and our results do not correspond to the reversal of postural inclination through the task. At last, we only suggest the effect of BS shape on posture and pressure points. The ideal or comfortable posture is changed according to aim in different clinical settings. Future long-term studies involving the unification of stroke onset or affected side are necessary.

## Conclusions

BS shape has direct effects on sitting posture. As compared with C-WC, the BS shape of P-WC ensures a neutral head and neck alignment, increases back contact area, and decreases high seat pressure in subacute stroke patients, possibly contributing to a reduction in postural or pressure asymmetry through movement because of continuous and stable pelvic support. BS shape needs to be considered by professionals when prescribing wheelchairs for ADL use.
